# Design, development and application of a compact robotic transplanter with automatic seedling picking mechanism for plug-type seedlings

**DOI:** 10.1038/s41598-023-28760-4

**Published:** 2023-02-02

**Authors:** Abhijit Khadatkar, A. P. Pandirwar, V. Paradkar

**Affiliations:** 1 ICAR-Central Institute of Agricultural Engineering, Bhopal, Madhya Pradesh 462038 India; 2 ICAR-Mahatma Gandhi Integrated Farming Research Institute, Motihari, Bihar 845429 India

**Keywords:** Energy science and technology, Engineering

## Abstract

Automation of agricultural operation such as seedling transplanting is needed to ensure efficient as well as timely operation. Robotics is the area that needs to be focused for the future of automatic seedling transplanter. This paper presents the design, development as well as working of the robotic transplanter (RT) for plug seedlings. The developed RT consists of three systems: (1) robot initiation; (2) seedling picking mechanism (SPM); and (3) vehicle movement system (VMS). The SPM consists of a main frame, manipulator, end-effector and control unit. Whereas, the VMS is having photoelectric sensor, robot controller and DC motor. The stepper motors were mounted on the main frame for movement in XY direction. The manipulator was on the crossbar that used to move the end-effector in Z-axis. The pick-up mechanism consists of an end-effector having jaw-type gripper controlled by servo motor. The control unit consists of microchip 16F877 and the system is controlled with computer programming. The gripper moves to each seedling in the pro-tray, grasp and pick-up the seedling, moves to the delivery point and then release the seedling. The manipulator was tested and analyzed for pickup and releasing of 96 seedlings with soil base from pro-tray. The initial experimental result showed that the seedling success rate, leakage rate and successful transplanting of 30 days old chilli seedling was 95.1%, 7.6% and 90.3%, respectively. Robotic technology seems to be expensive but the scope lies in the non-availability or high cost of manual labour and to ensure timeliness of repetitive field operations.

## Introduction

Application of robotics and artificial intelligence (AI) in agriculture is increasing rapidly with the use of hi-tech devices viz sensors, actuators, controllers etc. To achieve the desired goal i.e. transplanting of seedlings using robotics, an expert system needs to be integrated with the technology that can improve input use efficiency by doing the task with precision and accuracy. The main function of robotics is for targeting site-specific applications of inputs (seeds, fertilizers, pesticides, herbicides, water, etc.) as and when needed, at the right place, at right time and at right quantity.

AI uses techniques viz. speech recognition, visual perception, decision making, and language translations which can be enabled through computer systems to perform field operations which requires human intelligence^1^. The robotics and artificial intelligence are considered to be important aspect in automation of agricultural tasks. The major work was carried on various agricultural operation viz. navigation and object detection in real-time^2–4^; field crop monitoring using UAVs^5,6^; irrigation scheduling using mobile or web based applications from remotely^7^; detecting leaf diseases in crop as well as weeds in using vision-based algorithm^8,10,11^; livestock management in real-time^12–15^; computer based intelligent system^16–22^. Some application of AI and robotics were also reported in transplanting of seedling using vision or sensor based system. Robotic grippers, grasping as well as sensor-based methods and their applications have been reported for agricultural tasks^23^. Embedded system has been developed to automate the transplanting of vegetable seedlings in seedling transplanters^24,25^. Augmented use of electronics and computer application has made the working of robotic system possible for various field operations viz. transplanting, harvesting, and interculture, etc. for agricultural as well as horticultural crops^26^.

The transplanters available are categorized as semi-automatic vegetable transplanters (SVTs) and automatic vegetable transplanters (AVTs)^27^. Looking at the shortcomings of SVTs viz. labour demanding, lower efficiency, lower accuracy, etc., focus has been changed towards the growth of AVTs^28^. These AVTs uses electro-mechanical system for achieving its desired function. Some automatic system also used high end technology viz. sensors, vision based system, etc. giving to the rise of robotics into the transplanting operations. Transplanting seedlings by automatic means using robots is very useful where repetitive action is involved such as in case of seedling transplanting. These technologies seem to be expensive at first but, it will be compensated when one has to cover a large area in short time with high precision with use of human labour^24,27^. The basic function of the robot equipped with automatic mechanism is to identify the heathy seedling, pick-up the seedling from the pro-tray, move it to the desired location and then release it into the delivery point.

The work on robotic transplanter (RT) starts in 1980’s with the development of tractor mounted automatic pot seedling transplanter^29^. Some of the RT uses computer or machine vision system to integrate end-effector for simulating transplanting operation^30–35^. The RT that uses picking mechanism based on inserting needle/pin type structure into the soil base/plug of the seedling was developed and evaluated^36–47^.

A finger type mechanism was used for extracting seedlings from a nursery tray to plant pot using 4 fingers in the developed RT reported of using four inclined pin-type fingers to remove the seedlings from the nursery tray with the success rate was above 99% and transplanting capacity of 2800 pots/h^40^. In another study, Ma et al.^41^ reported that transplanting with developed prototype could be 90.71% qualified rate with transplanting frequency of 60 seedlings/min. A gantry structure was developed using pincette-type mechanism for automatic transplanting in greenhouse which can extracted 22 seedlings/min from a feeding tray with a success rate of 90%^42^. Han et al.^43^ indicated 90% success rate and 3% failure in discharging seedlings with two grippers which could extract 80 seedlings/min for tomato seedling. An intelligent transplanting system developed by Xin et al.^45^ reported success rate, leakage rate and transplanting frequency of 88.23%, 16.46% and 90 seedlings/min, respectively for pepper transplanting. It uses programmable logic controller (PLC) to detect whether the cell was empty or not, and to automate the movement of seedling pro-tray. This paper describes the design, development and evaluation of a RT by using programmable picking mechanism and sensor-based VMS to handle plug-type vegetable seedlings viz. chilli seedlings grown in pro-trays.

## Design of the transplanting robot

### Structure of the robotic transplanter (RT)

**Figure 1:**
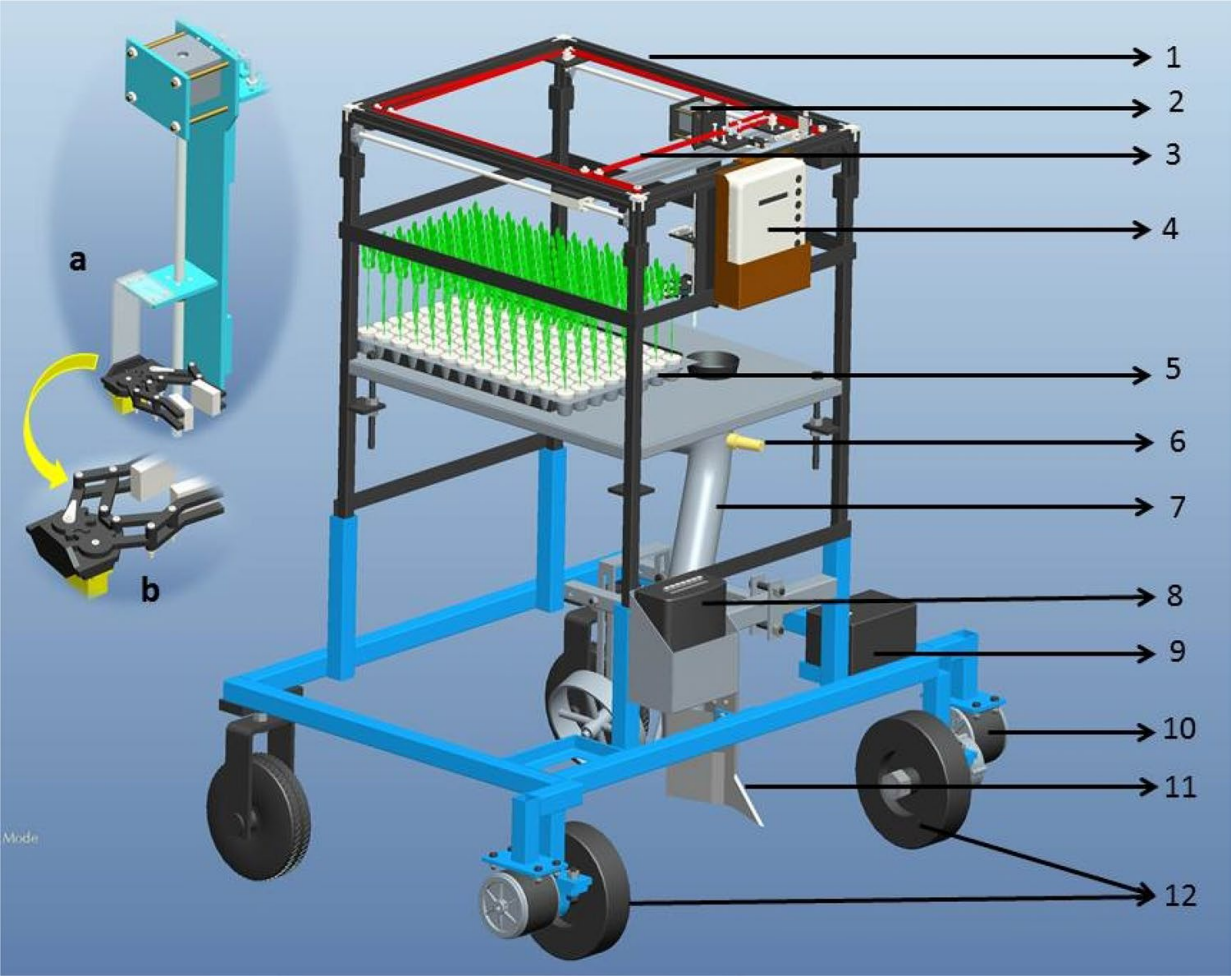
Robot transplanter. (**a**) Manipulator, (**b**) end-effector. (1-Main frame; 2-manipulator; 3-belt; 4-control unit for seedling pick-up; 5-seedling tray; 6-photoelectric sensor; 7-delivery tube; 8-control unit for robot; 9-battery; 10-dc motor; 11-furrow opener; 12-wheels).

The developed RT for plug seedlings is mainly composed of two mechanisms i.e. SPM and VMS (Fig. 1). The SPM is made up of belt and pulley attached on the main frame, stepper motor, manipulator, end-effector and controller. The VMS is consisted of photoelectric sensor, DC motors, stepper motors and controller. The controller system is composed of stepping motors, DC motors, photoelectric sensor, and programming controller. As soon as the robot starts, the manipulator moves to the seedling location and pick-up the seedling with servomotor actuated gripper. The end-effector then moves to its delivery point where the seedling gets dropped into the delivery tube. The photoelectric sensor placed on the delivery tube detects the seedling and actuate the VMS to move to the next location after delivering the seedling into the furrow. Here, the seedling tray is stationary and manipulation moves in XY axis for extracting the seedling from the protray as per predefined path. A pair of stepper motors attached on the main frame moves the manipulator in XY direction whereas, the end-effector moves in Z axis. A furrow opener was used to make a furrow in the soil. As the seedling dropped into the furrow, the press wheel attached next to the deliver pipe compact the soil around the seedling. The specification of the developed RT is shown in Table 1.

**Table 1:** Specification of the developed robotic transplanter.

S. no	Component	Specification
1	Structure	Machine frame for X, Y, Z Size (l × b × h): 630 × 510 × 120 mm (effective working area 420 × 297 mm) Z-axis mechanism for gripper mounting (stepper motor with T8 screw)
2	Seedling picking mechanism (SPM)	Stepper motor Stepper motor drive SMPS power supply Limit sensor set Gripper for z-axis Control unit
3	Vehicle movement system (VMS)	250 W geared DC motor 24 V/360 rpm Full load current: ≤ 13.4A Control circuit with photoelectric sensor

### Working principles of RT

**Figure 2:**
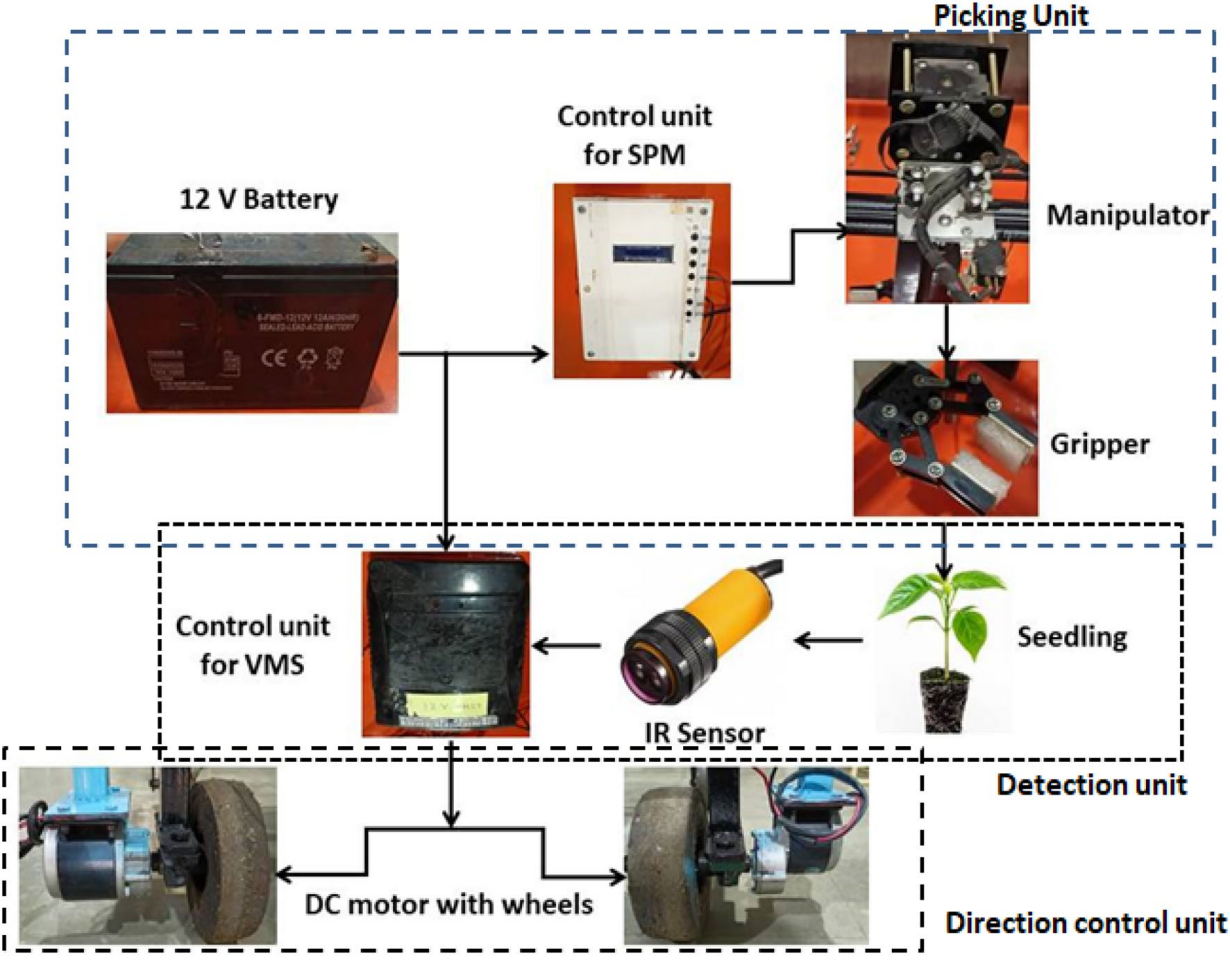
Working principle of the robotic transplanter (**a**) picking unit; (**b**) detection unit; (**c**) direction control unit.

The working principle for RT was illustrated in Fig. 2. The RT works on the 12 V battery supply. As soon as the programme initiate, the first part of the system i.e. SPM starts working. The manipulator moves to the XY (0,0) initial point i.e. delivery point. The programme in the controller then needs to be set as per the requirement viz. speed, pulse, no. of row and columns seedling, etc. As the programme is set in the controller and then it needs to start the programme through start button. Now the manipulator moves to the 1st seedling, the gripper grasp the seedling and moves upwards in Z axis till it come above the pro-tray i.e. 45 mm above. The manipulator now moves to the delivery point XY (0,0), where the gripper opens and release the seedling into the furrow through delivery pipe. After releasing the seedling, the manipulator moves to the 2nd seedling and the process continues till the last seedling was delivered. The gripper opens and closes with help of servomotor attached on it. As soon as the seedling dropped into the delivery pipe, the second part i.e. VMS starts working. The photoelectric sensor placed in the delivery pipe detects the seedling and moves the robot forwards to the next location, and the process continues till the last seedling was picked and delivered. The motion of the robot can be controlled by varying the speed at the controller. In this way, the whole process of seedling pick-up and planting continues.

## Design of the SPM and VMS

### Design requirements

The structure of developed SPM was shown in the Fig. 1a. To transplant plug seedlings, the SPM was developed in such a way to simulate the gripping of seedling in accordance with the human hand from the pro-tray and then releasing it at delivery point i.e. XY (0,0). By investigating the features of the SPM, to pick-up the seedling successfully from the pro-tray, it should meet the following design requirements:

1. The manipulator should follow the straight path to reach the seedling location and seedling to be picked up and back to the initial point.
2. The end-effector (Fig. 1b) should open and close completely to hold the seedling firmly without damage.
3. The length of path of seedling pick-up needs to ensure that it is not obstructed with the protray.
4. The seedling should be released exactly above the delivery tube and as straight as possible into the delivery pipe.

The VMS is used to move the robot to the next location after dropping the seedling into the furrow. As soon as the seedling dropped into the delivery pipe, the robot should actuate and move to the next dropping point. In order to move the robot forward, the design of the VMS should meet the following requirements:

1. The photoelectric sensor should identify the dropped seedling into the delivery pipe.
2. Controller should immediately respond to the photoelectric sensor and move the complete robot to the next location as defined in the programme.

### Structure and working principle of SPM & VMS

**Figure 3:**
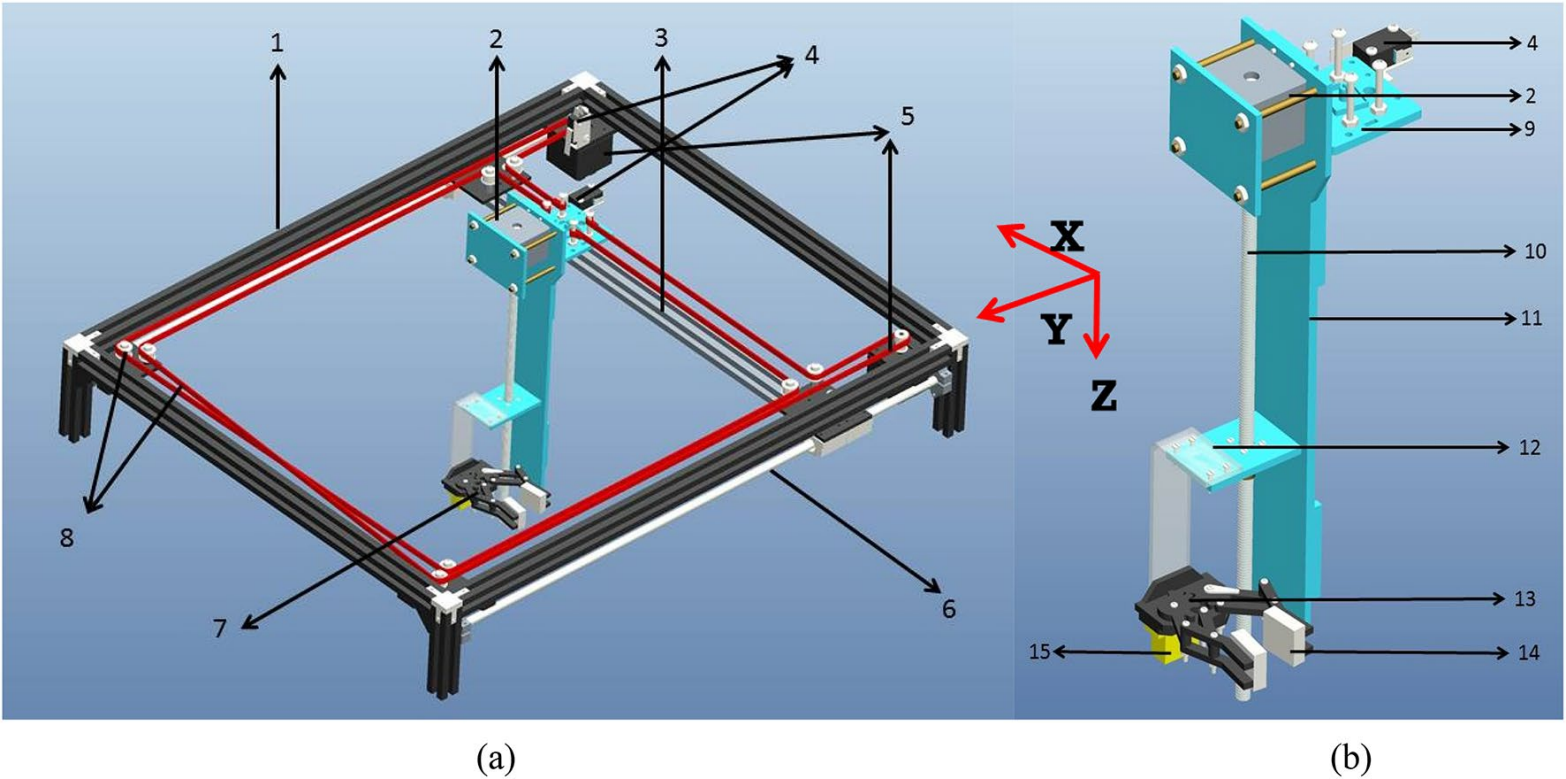
(**a**) Seedling pick-up mechanism, (**b**) Manipulator. (1-Main frame; 2-manipulator; 3-cross bar; 4-electronic switch; 5-stepper motors; 6-horizontal shaft; 7-end-effector; 8-belt-pulley arrangement; 9-support plate; 10-screw shaft; 11-sliding plate; 12-end-effector support plate; 13-gripper; 14-cushion pad; 15-servomotor).

The design adopted to realize the SPM and manipulator for automatic extraction of plug seedling, as is illustrated in Fig. 3. The SPM is mainly comprising of a main frame, stepper motor with screw shaft, belt-pulley arrangement, gripper, servomotor and electronic switch.

**Figure 4:**
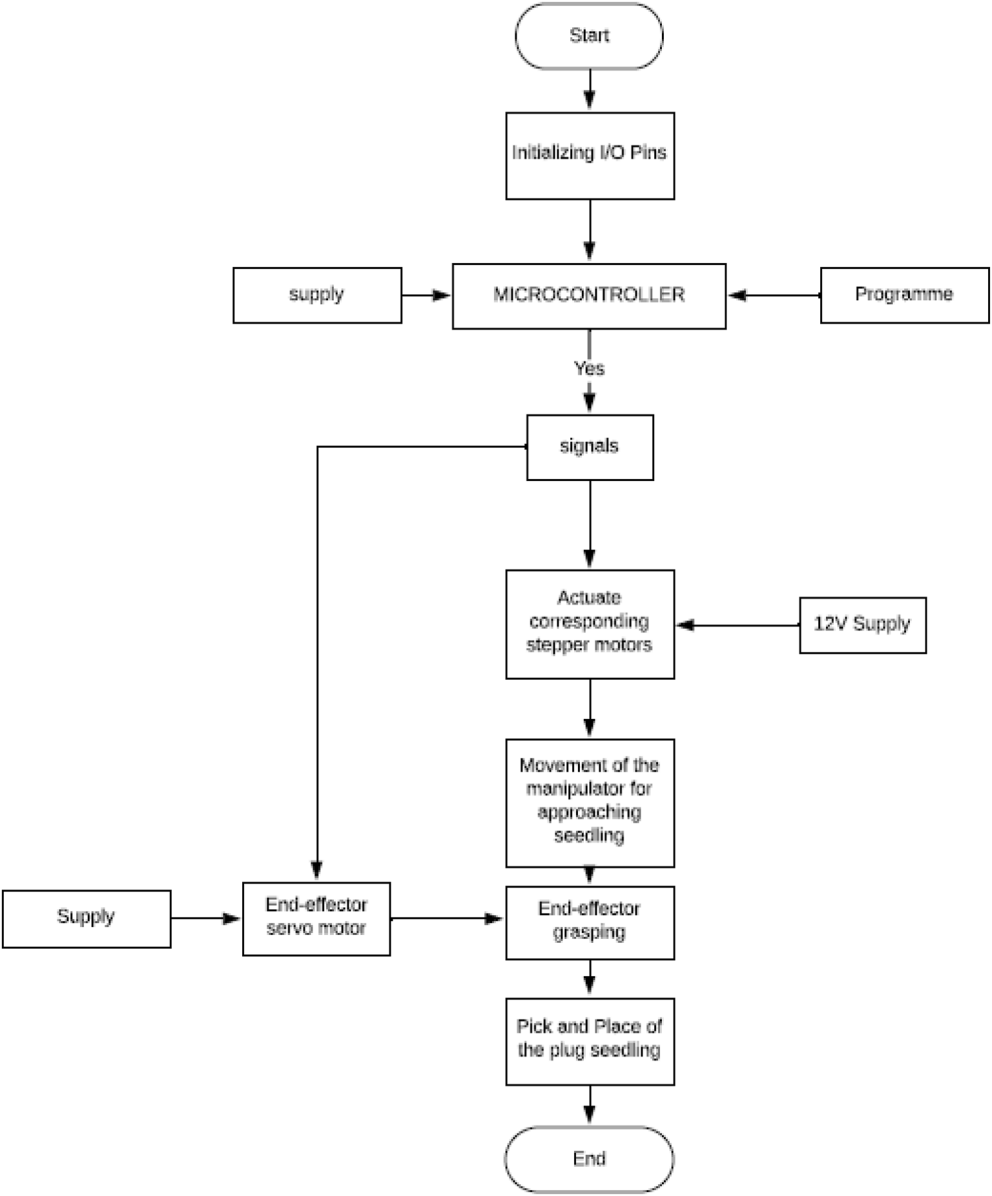
Flow diagram of the seedling picking mechanism used in the robotic transplanter.

As mentioned in “Working principles of RT” section, the manipulator starts moving in XY plane on the main frame (Fig. 3) and the end-effector moves in Z axis on stepper motor screw shaft. The gripper used to grip, hold and release the seedling into the delivery pipe through controlling angles of servomotor. The manipulation moving in XY plane extract the seedling from the pro-tray as per predefined path. The flow diagram of the seedling picking mechanism used in the robotic transplanter is shown in Fig. 4.

The main function of the VMS is to move the complete unit i.e. robot forward to the next planting location. As soon as the photoelectric sensor detects the seedling, the controller then actuates the robot to move forward as per the predefined programme. In this way, the whole process of planting continues again and again.

## Design of control system hardware circuit of RT

### Design of control system hardware circuit for SPM

**Figure 5:**
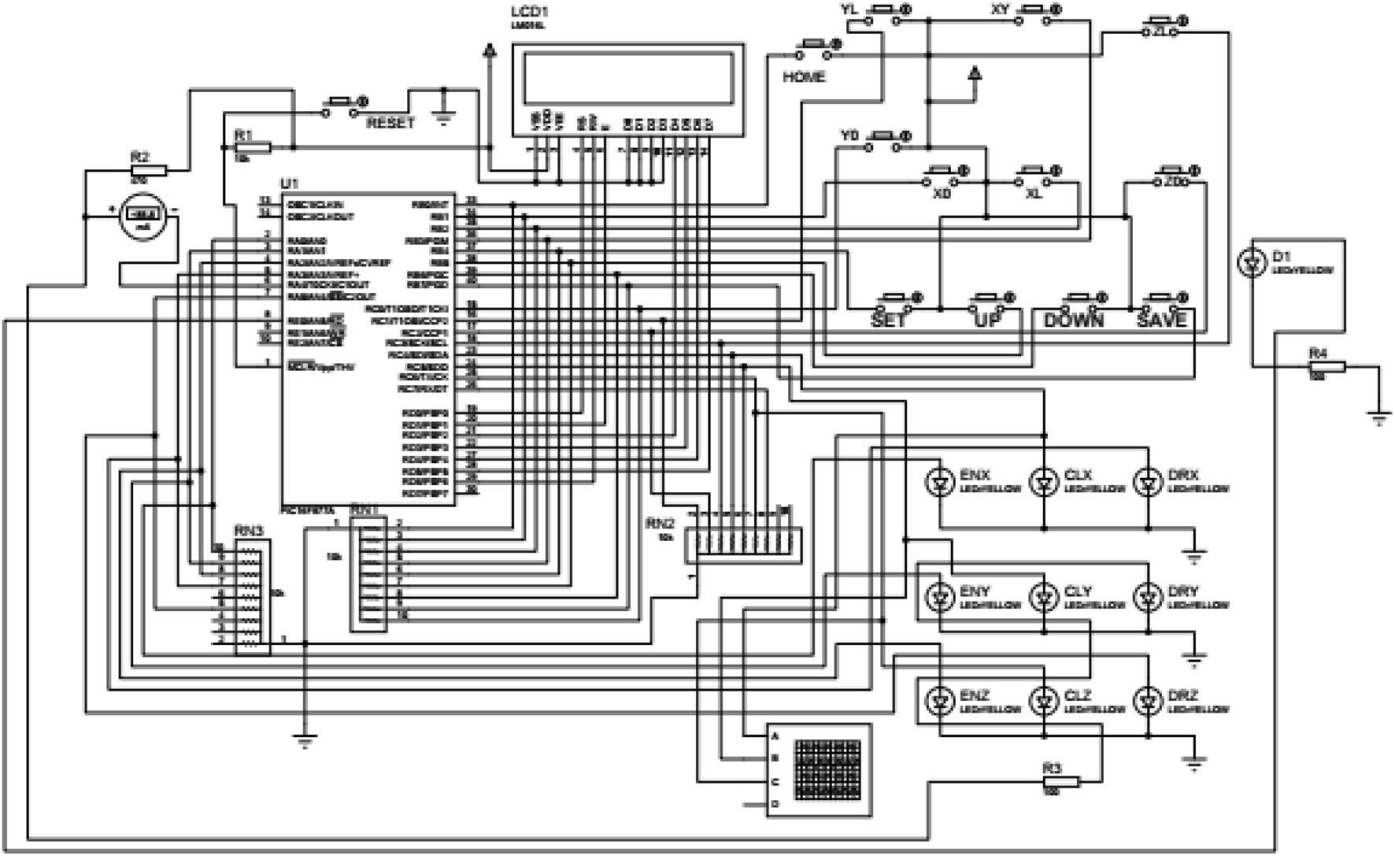
Circuit diagram of the developed seedling picking mechanism (SPM).

To design the circuit of the RT i.e. SPM, it is essential to detect the location of each seedling placed in the pro-tray and also the delivery point i.e. XY (0,0). The mechanism works on power supply from 12 V battery, as is shown in Table 2. Among them, the stepping motors, manipulator, servomotor and PLCs are driven by a 12 V battery, as is shown in Fig. 5. The input terminals of the PLC are coupled with the electronic switch, the manipulator and end-effector.

**Table 2:** Main hardware design used in the robotic transplanter.

Items	Name	Type	Voltage, Current	Number
Signal acquisition	Photoelectric sensor	E18-D80NK	5 V, 300 mA	1
	Electronic switch	Micro-switch 12 mm		2
Control unit	Micro-chip 16F877 based PLC	8-bit	12 V, 3A	1
Driving element for SPM	Stepping motor	NEMA 23 10.1 kg-cm	12 V	3
	Servomotor based manipulator	TowerPro SG90	3.0–7.2 V	1
Driving element for VMS	DC motor	MY1016Z2 (250W 360 rpm Geared)	12 V, ≤ 13.4A	2
	Wireless remote control	TPS-WLPower Pro	12 V	1
Power supply	Battery	Lead acid, SMF	12 V, 40A	1

The electronic design of SPM consists of a microcontroller (PIC16F877A), stepper motor and driver, power supply, limit sensor set, gripper and LCD Screen. Five switches were provided for adjusting the picking and placing position of the gripper viz. set, up, down, save and reset. When all the switches press according pre-position data of seedling was sent to the controller and with the same switch this data is saved in the EEPROM memory of the controller. After finishing the sequence, the controller provides suitable signals to stepper motor driver to move stepper motors accordingly so that the picking of the seedling can be accomplished but whenever the seedling was dropped in the delivery tube, IR sensor sends signal to the controller so that the gripper can move to the next position of the seedlings on the portray. And the same sequence is continued till the last seedling of the portray is completed.

### Design of hardware circuit control system for VMS

**Figure 6:**
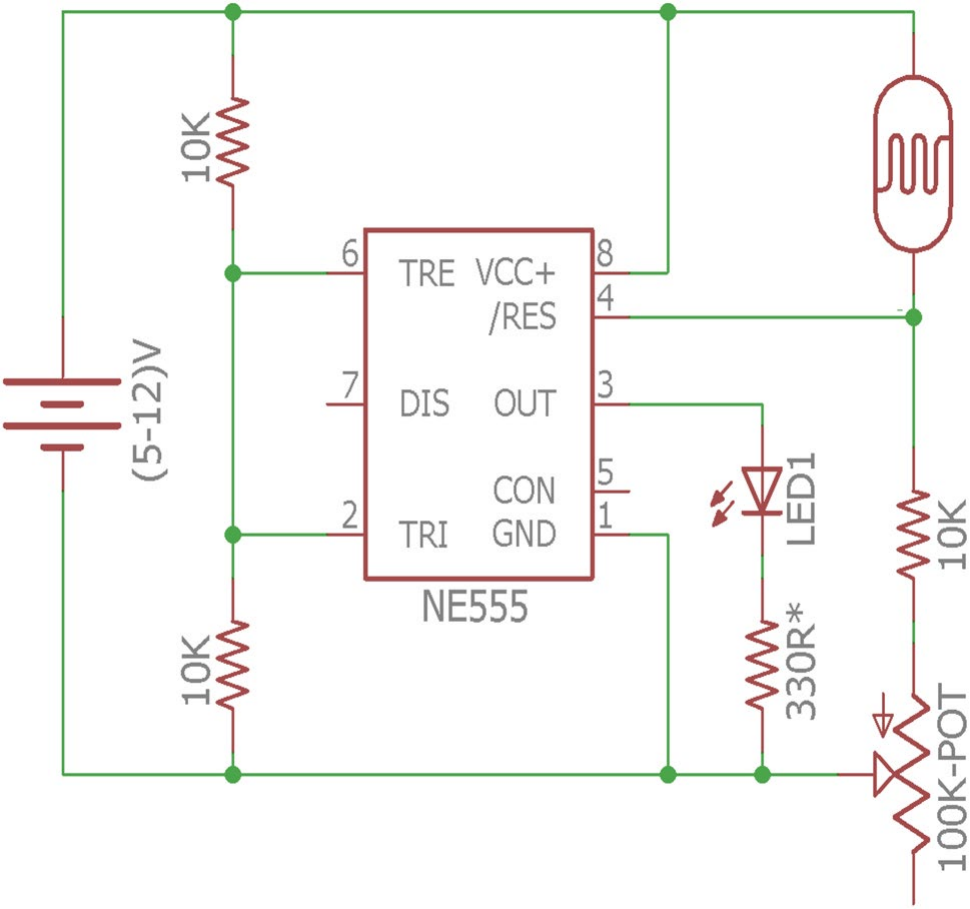
Circuit diagram of vehicle movement system.

The movement of the vehicle can also be controlled remotely using wireless signal transmission. In this system one wireless remote controller and single computer chip is used which receives signal from controller and control the revolving direction of the propelling DC motor which enables the movement of vehicle in either forward or backward direction. The circuit design of the VMS is shown in Fig. 6. The sensors are connected to the input of PLC as per the functional requirements.

### Design of control system process

**Figure 7:**
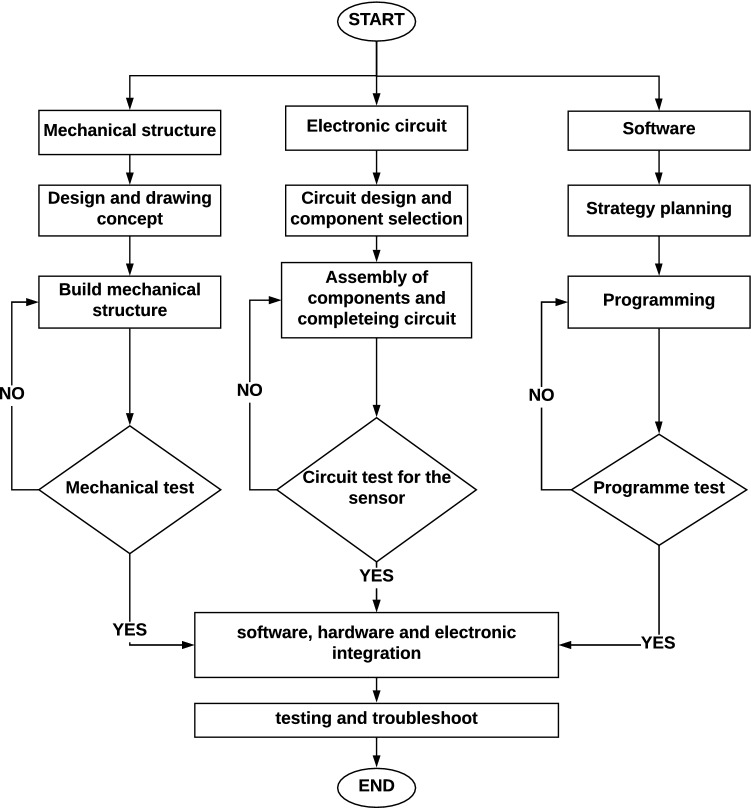
Flow chart of the complete robotic transplanter.

As per the requirements, the main function of the RT is to pick-up the seedling from the pro-tray and releases them into the furrow during operation. The complete flow chat of the system was shown in Fig. 7. According to the working principle of the RT, each action is distributed into some sections: the first is seedling extraction and releasing; and the second part is VMS with furrow making planting followed by soil covering. In transplanting operation, each mechanism should work independently and also one after the other in a synchronized manner with each other and without any interruption. The synchronization of each unit is described below:

1. Operational coordination among the seedling picking part, the manipulator moves to the 1st seedling position, the gripper grasp the seedling lift it up and moves back to (0,0) position and then release the seedling. During seedlings pick-up, the pro-tray is stationary and manipulator moves until the process completes. After the removal of first seedling from the pro-tray till it gets released into the delivery pipe, the robot is stationary.
2. Working coordination between the seedling releasing parts and the VMS part of the RT. The forward speed of the vehicle should not be more than the seedling picking to delivery speed to meet the requirements of plant spacing.

This RT is a single-row unit, which can transplant one plug seedlings at a time. The SPM of this machine has one gripper.

**Figure 8:**
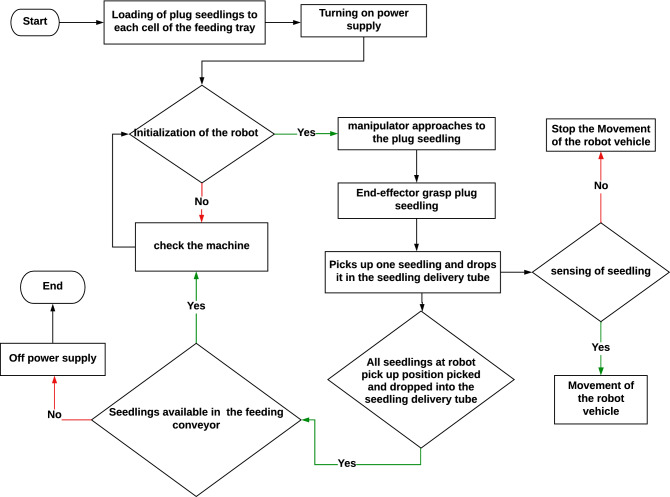
Flow chart of the overall control process of the robotic transplanter.

The overall control process of the transplanting machine was designed, as per the functional requirements of each mechanism to attain the synchronized movement among them, as illustrated in Fig. 8. As soon as the start button was pressed, the manipulator moves to the 1st seedling, the gripper mounted on the end-effector pick-up the seedling and release it into the furrow through delivery pipe. After the seedling extraction part completes the VMS part starts working. The photoelectric sensor detects the seedling and robot moves to the next location as per requirement fed to the programme.

## Result and discussion

Some physical parameters of the seedlings, physical dimension of the seedlings i.e. height, weight and stem diameter of the seedling were considered for the designing the robot. The average height, weight and stem diameter of the seedling were 116 mm, 13.1 g and 0.1 mm, respectively. The breaking pressure, compressive force and rupture force were also measured. The breaking pressure was very negligible so it cannot be measured as the stems are more susceptible to the failure during compression. So, the compression force measured using Universal Testing Machine (UTM) was 144.98 (± 29.3) N. However, the rupture force required for 30 days old chilli seedlings was 12.7 N. The rupture force of the seedlings was measured with Texture Analyser (Make: Stable Micro System, UK).

The main factor for seedling picking rate are moisture content of root media and force required to extract the seedling from the portray. The moisture content of the root media of the seedling at the time of transplanting ranged between 55 and 75% (wb). The average force (adhesion) required for the seedling picking/ extracting from the portray was 0.95 (± 0.22) N. As the force required to lift the seedling was very less, this manipulator was tested several times for picking seedlings and it was observed that damage to the rhizomes were negligible as it is not contacting directly to the rhizomes.

The working performance of the developed RT for transplanting plug seedlings was evaluated under ambient condition. The seedling variety of the chilli used was Pusa Jwala. The chilli seedlings were grown in 96 cell seedling pro-trays, and the filling composition was made up of coco-peat, vermiculite and perlite in a ratio of 3:1:1. The filling composition was covered with vermiculite and perilite after sowing the seeds. The moisture content was kept between 45 to 60% of the seedlings. About 30 days old seedlings with 4–5 leaves was used for testing, the average seedling height is about 96 mm. Dummy seedling of similar shape and size was used to compare the performance. Seedling properties are very important while designing the RT for plug seedlings^48^.

The success rate of SPM, leakage rate and successful transplanting are the essential indices to evaluate the transplanting performance of the developed RT and estimated as per the investigators^45,49^:

1 $${\text{S}} = \left( {{\text{N}}/{\text{N}}_{0} } \right) \times {1}00\%$$

2 $${\text{L}} = \left[ {{1} - \left( {{\text{N}}_{{1}} /{\text{N}}_{0} } \right)} \right] \times {1}00\%$$

3 $${\text{T}} = \left( {{\text{F}}/{\text{N}}_{0} } \right) \times {1}00\%$$

where S = overall success rate; L = leakage rate; T = successfully transplanting; N_0_ = the total number of seedlings; N = the number of seedlings picked up; N_1_ = the total number of seedlings released; F = the number of seedling successfully transplanted into the furrow with proper soil compaction and seedling inclination less than 30°.

**Figure 9:**
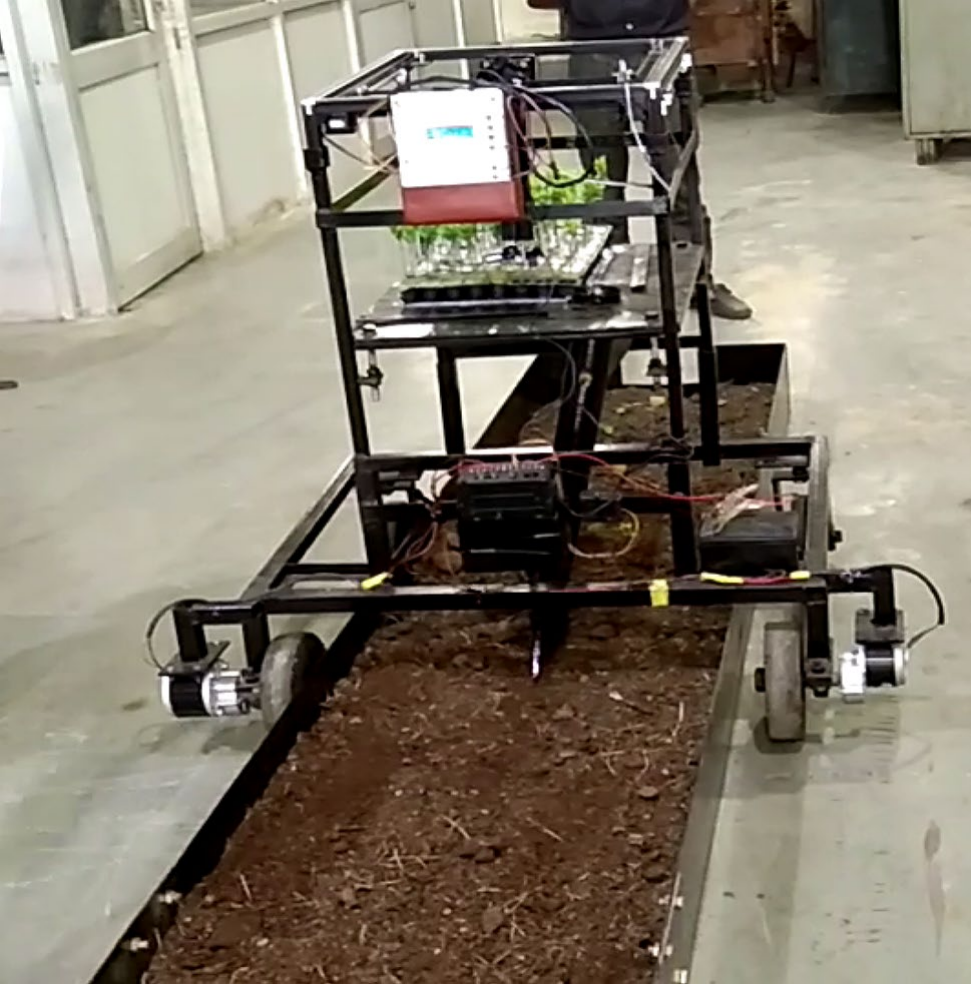
Test setup of robotic transplanting with plug seedlings.

The seedling pro-tray was placed on the transplanter platform. The system was calibrated for the set programme on PLC and control system was started. The test setup was shown in Fig. 9. As soon as the system start, the manipulator moved to the 1st seedling of the first row, the servomotor at the gripper pick-up the seedling and delivery it to the delivery tube. Finally, the movement of manipulator stops at the dropping point by pressing the electronic switch and the seedling gets released into the furrow. The photoelectric sensor detects the seedlings in the delivery tube and move the vehicle forward as per the developed programme. The test data on success rate, leakage rate and successful transplanting were calculated and evaluated.

The results obtained during testing were presented in the Table 3. The seedlings picked up successfully means the seedlings that are picked up without any physical damage to root and plant. The seedlings released successfully means the seedlings that are dropped into the delivery pipe without any prominent damage. From Table 3 it was observed that the seedling picking rate and leakage rate was 98.6% and 1.7%, respectively with dummy seedlings. Whereas, the seedling picking rate and leakage rate for 30 days old chilli seedling was 95.1% and 7.6%, respectively. Also, the successful transplanting with dummy and chilli seedling was 97.2% and 90.3%, respectively. As the dummy seedlings were more straight and sturdy, the seedling picking rate and successful transplanting were higher as compared to chilli seedlings.

**Table 3:** Test performance of the developed robotic transplanter.

Seedling type	Total number of seedlings	Number of seedling picked up	Number of seedling dropped	Number of seedling transplanted in furrow	Success rate (%)	Leakage rate (%)	Successful transplanting (%)
Dummy seedling	96	96	95	95	100.0	1.0	99.0
	96	95	95	95	99.0	1.0	99.0
	96	93	93	90	96.9	3.1	93.8
**Mean**					**98.6**	**1.7**	**97.2**
Chilli seedling	96	90	86	84	93.8	10.4	87.5
	96	91	90	88	94.8	6.3	91.7
	96	93	90	88	96.9	6.3	91.7
**Mean**					**95.1**	**7.6**	**90.3**

Significant values are in bold.

Based on the test result, there are some suggestions to enhance the performances of robotic transplanting device of plug seedlings: increasing the gripper opening, decreasing the length of seedling picking, and reducing travel time from pick-up point to delivery point.

## Conclusions

A compact robotic transplanting machine with automatic seedling pickup mechanism and vehicle movement system was designed and developed to meet the need of small and marginal vegetable growers. The following are main findings of the study:

1. The developed RT can successfully extract and transplant seedling into the furrow and the transplanting cycle to transplant the seedling 20 s.
2. The RT was tested with 96 seedlings grown in portray and the seedling picking success rate, leakage rate and successful transplanting of 30 days old chilli seedling was 95.1%, 7.6% and 90.3%, respectively.
3. The RT can efficiently adapted by used by the marginal farmers for vegetable cultivation in playhouse’s or shed nets.
4. The robotic technology may seem to be expensive but the scope lies in the non-availability or high cost of manual labour and to ensure timeliness of repetitive field operations.

## Data Availability

The datasets used and/or analyzed during the current study are available from the corresponding author on reasonable request.

## Abbreviations

RT: Robotic transplanter
DC: Direct current
SVTs: Semi-automatic vegetable transplanters
AVTs: Automatic vegetable transplanters
SPM: Seedling picking mechanism
VMS: Vehicle movement system
SMPS: Switch-mode power supply
PLC: Programmable logic controller
